# Contrast-enhanced ultrasonography of the pancreas in healthy cats

**DOI:** 10.1186/s12917-015-0380-2

**Published:** 2015-03-17

**Authors:** Alessia Diana, Nikolina Linta, Mario Cipone, Valeria Fedone, Joerg M Steiner, Federico Fracassi, Annamaria Grandis, Marco BaronToaldo

**Affiliations:** Department of Veterinary Medical Sciences, Alma Mater Studiorum – University of Bologna, Via Tolara di Sopra 50, I-40064 Ozzano Emilia Bologna, Italy; Freelance sonographer, Messina, Italy; Department of Small Animal Clinical Sciences, College of Veterinary Medicine and Biomedical Sciences, Texas A&M University, College Station, TX USA

**Keywords:** Cat, Contrast-enhanced ultrasonography, Diagnostic ultrasound, Pancreas, Sonovue

## Abstract

**Background:**

This study describes the pattern of ultrasonographic contrast enhancement of the pancreatic body and left lobe using a second-generation commercial contrast medium (Sonovue) in 10 clinically healthy cats.

**Results:**

Following contrast medium administration, microbubbles were observed within the splenic artery. This was followed by an inflow of contrast medium into the pancreatic capillary beds, providing a uniformly contrast-enhanced pancreas at peak intensity (PI). At the time of PI, a replenishment of the splenic and portal veins started and increased progressively during the wash-out phase. During the wash-out phase, the echogenicity of the pancreatic parenchyma decreased progressively. Perfusion parameters included arrival time (4.69 ± 1.26 s), time to peak from injection (7.52 ± 1.88 s), time to peak from initial rise (2.84 ± 0.88 s), peak intensity (6.58 ± 2.66 a.u.), and wash-in rate (2.11 ± 1.79 a.u./s).

**Conclusions:**

This perfusion pattern of normal pancreatic parenchyma may be useful for characterising cats with exocrine pancreatic disorders.

## Background

Abdominal ultrasonography can be a useful diagnostic tool for the work-up of cats with suspected pancreatitis. The ultrasonographic appearance of the normal feline pancreas and its age-related changes have been described [1-3]. Reference ranges for the thickness of the pancreatic body, lobes and duct have also been reported [4]. A variety of ultrasonographic changes (i.e., diffuse hypoechogenicity, pancreatic enlargement, hyperechoic peripancreatic mesentery and peritoneal effusion) have been described in cats with pancreatitis [3,5,6], although the sonographic features can overlap with other exocrine pancreatic abnormalities [7-9]. The reported sensitivity of abdominal ultrasonography for the diagnosis of feline pancreatitis ranges from 20% to 67% [5,10,11]. This low sensitivity suggests that imaging of the inflamed pancreas is more difficult in cats than in dogs, or that the ultrasonographic appearance of pancreatitis in cats differs from that reported in dogs [8].

In human medicine, contrast enhanced ultrasonography (CEUS) is widely applied for identification of pancreatic tumours based on their pattern of vascularisation [12-16]. Furthermore, CEUS accurately detects pancreatic necrosis, helping to predict the clinical outcome of patients with acute pancreatitis [17,18].

There are only a few reports concerning the use of CEUS for the evaluation of the pancreas in cats [19,20] and dogs [21-23]. In cats, one study described the quantitative CEUS analysis of perfusion in different abdominal organs of healthy cats, including the right pancreatic lobe [20]. Another study described contrast-enhanced and colour Doppler ultrasonography of the pancreas in healthy and diseased cats using a first generation contrast agent [19]. The pancreatic perfusion in healthy dogs using either a single bolus injection [21] or continuous infusion [22], and in dogs with cerulein-induced acute pancreatitis [23] and with pancreatic tumours [24] were also reported.

The first aim of the present study was to describe CEUS patterns of the body and the left lobe of the pancreas in clinically healthy cats. The second aim was to evaluate the thickness of the pancreatic left lobe both before and after injecting the contrast medium.

## Methods

### Study population

Ten clinically healthy client-owned adult cats were used in this study. Cats represented four breeds including European short hair (4), Maine Coon (3), Norwegian (2) and Persian (1). There were three intact males, two neutered males, four intact females and one neutered female. The age of the cats ranged from 2 to 9 years (mean ± SD, 5.5 ± 2 years), and the cats’ bodyweight ranged from 4 to 8.4 kg (mean ± SD, 5.3 ± 1.5 kg).

Cats were considered clinically healthy and had no history of hepatobiliary, pancreatic or gastrointestinal disease for the previous 12 months. Physical examination, complete blood count, serum biochemical analysis including feline trypsin-like immunoreactivity (fTLI) and feline pancreatic lipase immunoreactivity (fPLI) concentrations, urinalysis and faecal examination for intestinal parasites were normal. All cats were also negative for feline leukaemia virus antigen and feline immunodeficiency virus antibody. Informed owner consent was obtained and all procedures were approved by the Ethical Committee of the University of Bologna (Authorisation reference number: 17/72/2012; date of approval 10-01-2012).

### Ultrasound imaging

**Figure 1:**
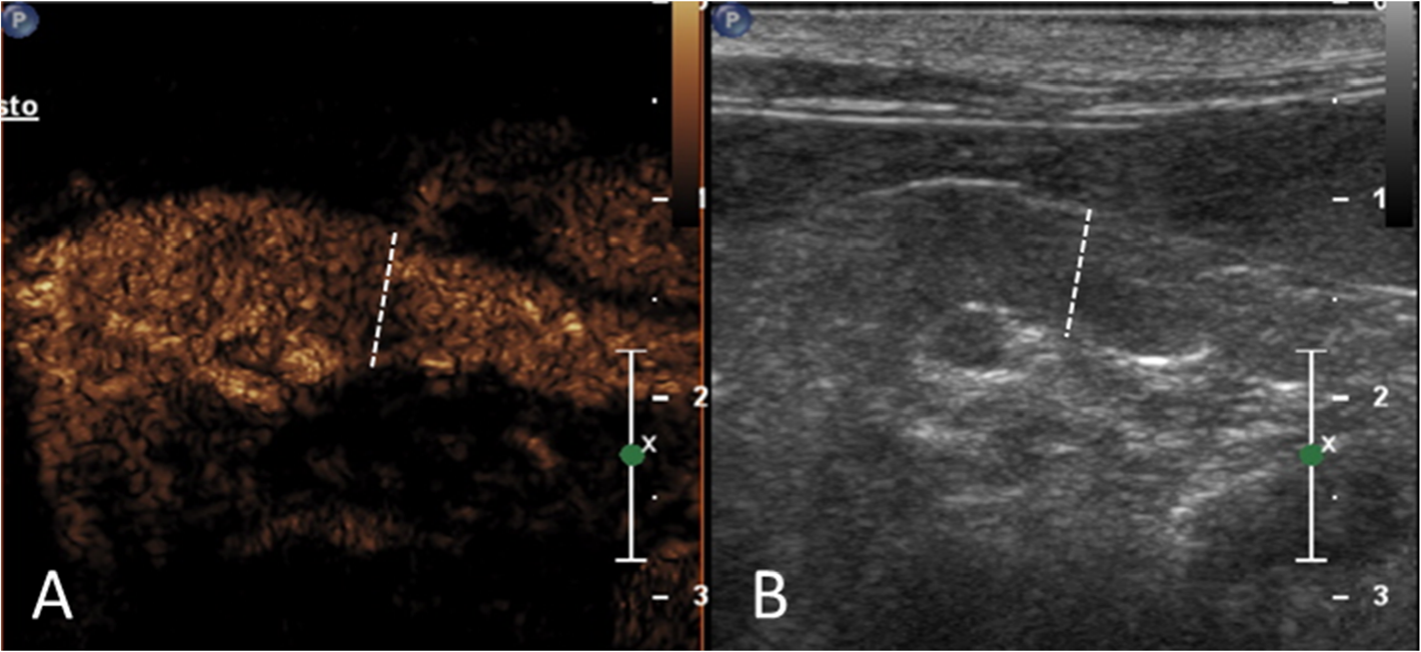
**Longitudinal section of the pancreatic body and left lobe of a healthy cat in the contrast enhancement image (A) and the grey scale image (B), respectively.** An example of left lobe thickness measurements is showed. The white dotted line represents the thickness of the left pancreatic lobe at peak enhancement.

Food was withheld overnight (at least 12 h) prior to imaging. All procedures were conducted by the same ultrasonographer (AD), using a real-time ultrasound machine^a^ equipped with a broadband curved array transducer (5–8 MHz) and a linear array transducer (3–9 MHz). Hair over the abdomen was clipped, the skin surface was cleaned with 70% isopropyl alcohol, and coupling gel was applied. The cats were awake and were restrained manually during the examination. With each cat in dorsal or right lateral recumbency, the body and the left lobe of the pancreas as well as the portal vein were identified for CEUS using a subcostal approach. An image of the longitudinal section of the pancreatic body and left lobe was obtained and the transducer was not moved subsequently (Figure 1). Contrast-specific software (Pulse Inversion Harmonic and Power Modulation combined – PMPI) with a low mechanical index set (0.07) was activated. The gain setting was regulated to obtain anechoic pancreatic parenchyma at baseline, and a focal zone was placed just below the pancreas to minimise microbubbles destruction. These CEUS settings were tested and standardised in our ultrasound laboratory and optimised for the ultrasound machine, probe and application used in this protocol.

The contrast medium^b^ was prepared and injected by the same operator, using the following standard procedure. The microbubble dispersion was prepared before use by injecting through the septum 5 mL of sodium chloride 9 mg/mL (0.9%) solution for injection to the contents of the vial. The vial was then shaken vigorously for a few seconds until the lyophilisate was completely dissolved. Four mL volume of the dispersion was drawn into a syringe. Just before drawing into the syringe, the vial was agitated to re-suspend the microbubbles. The contrast medium was administered manually through an indwelling cephalic venous 22G catheter as a rapid bolus of 0.5 mL, followed immediately by a rapid bolus of 4 mL saline.

The images were recorded as cine-segments in DICOM format of 60 s starting from contrast medium injection, and were transferred to a personal computer.

Show Case software^c^ was used to view the images and to export selected frames for qualitative analysis. The distribution of the contrast medium enhancement within the pancreatic lobe was evaluated subjectively as satisfactory or unsatisfactory, based on the degree of parenchymal enhancement and homogeneity at peak intensity (PI). The CEUS pattern of the pancreatic body and left pancreatic lobe were evaluated during contrast uptake (wash-in phase), at PI and during progressive wash-out of contrast medium. Contrast medium inflow at the splenic vessels (both artery and vein) and at the portal vein was also evaluated.

**Figure 2:**
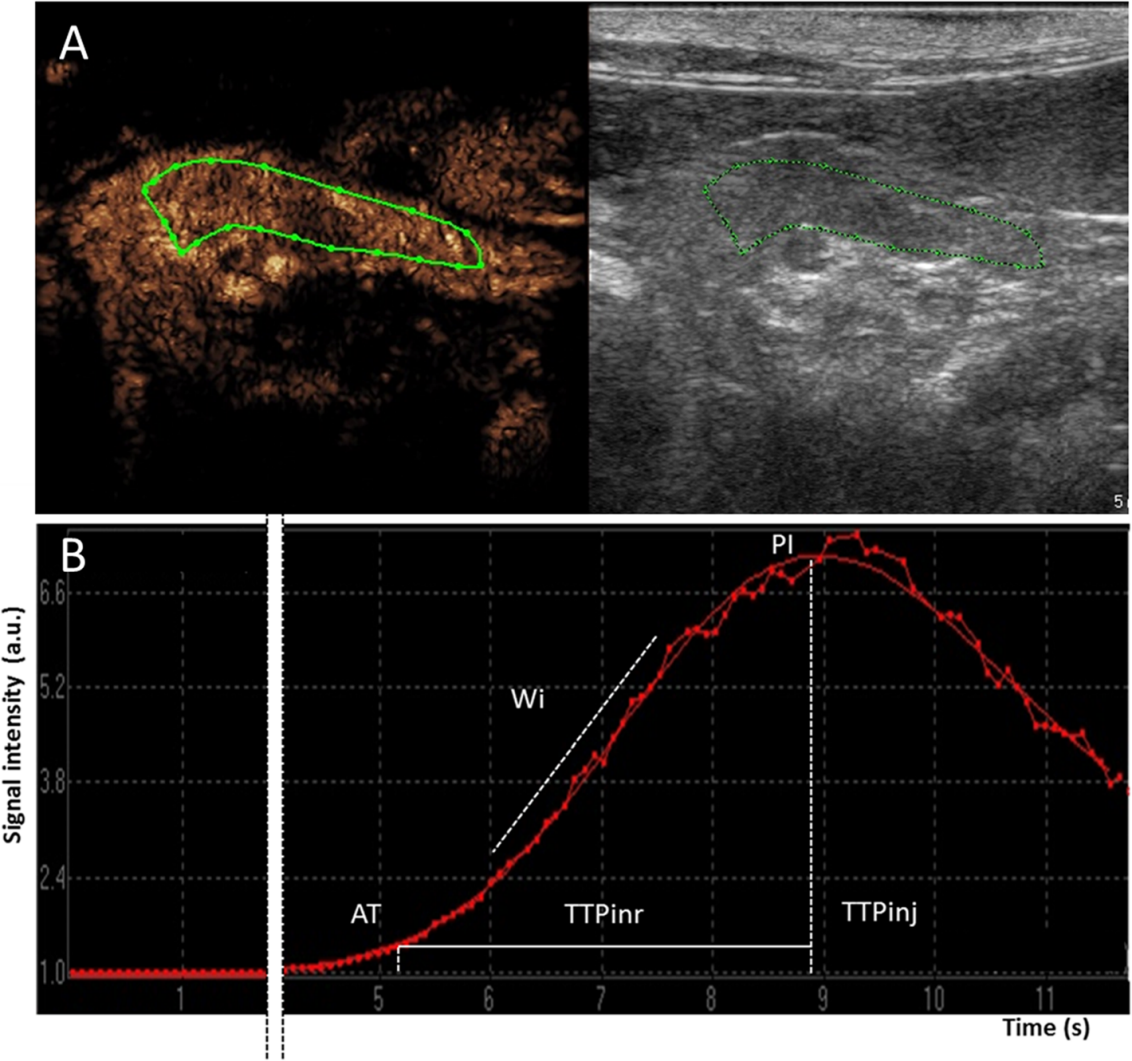
**Contrast enhancement image and the grey scale image (A) and signal intensity in arbitrary units (a.u.) as a function of time in seconds (s) (B) of the pancreatic body and left lobe in a healthy cat.** Region of interest is delimited by the green line. The red dotted line represents the raw data derived from the quantification. The continuous red line corresponds to the curve given automatically by the software using a proprietary algorithm. The image is cut on purpose between 1 and 5 s for formatting reasons. AT, arrival time; TTPinr, time to peak from initial rise; TTPinj, time to peak from injection; PI, peak intensity; Wi, wash-in rate.

A commercial software program^d^ was used for quantitative computerised analysis of the contrast medium blood pool phase. A region of interest (ROI) covering the body and left pancreatic lobe as much as possible was manually drawn to avoid adjacent major vessels (Figure 2). The ROI was maintained in the same position by the motion compensation tool of the software. This tool prevents the displacement of the ROI during respiratory motion. Furthermore, the ROI was adjusted manually on those frames severely affected by respiratory motion. Artifactual data from adjacent tissue that moved into the ROI during respiratory motion were removed manually from the final data set to reduce noise. The raw data obtained from each cat were plotted in quantitative time-intensity curves after fitting of a mathematical algorithm. Only fit-curves with quality of fitting expressed as *r*^*2*^ equal or more than 0.9 were used for subsequent analysis. The following perfusion variables were recorded: arrival time (AT, expressed in s), defined as the time when the contrast signal increases to greater than double the baseline value in the time-intensity curve; time to peak from injection (TTPinj, expressed in s); time to peak from initial rise (TTPinr, expressed in s); peak intensity (PI, expressed in arbitrary units [a.u.]); and wash-in rate (Wi, expressed in a.u./s), defined as the slope of the curve during the wash-in phase (Figure 2). Wash-in was calculated as the maximal change rate, using data points 10% above baseline and 10% below peak to exclude variability at the toe and shoulder of the time-intensity curve, respectively. Using an electronic calliper, the thickness of the left pancreatic lobe was measured by dual imaging mode from the grey scale image before contrast medium injection and from the contrast image at peak intensity, respectively (Figure 1). The mean value of three measurements for each cat was used for analysis.

### Statistical analysis

The data were then regressed for significance of linearity using a D’Agostino-Pearson test and expressed as means and standard deviations (SD). A two-tailed Student’s *t* test was used to compare the thickness of the left pancreatic lobe before and after contrast medium injection. A *P* < 0.05 value was considered statistically significant. Statistical analyses were performed using a commercial software package^e^.

## Results

**Figure 3:**
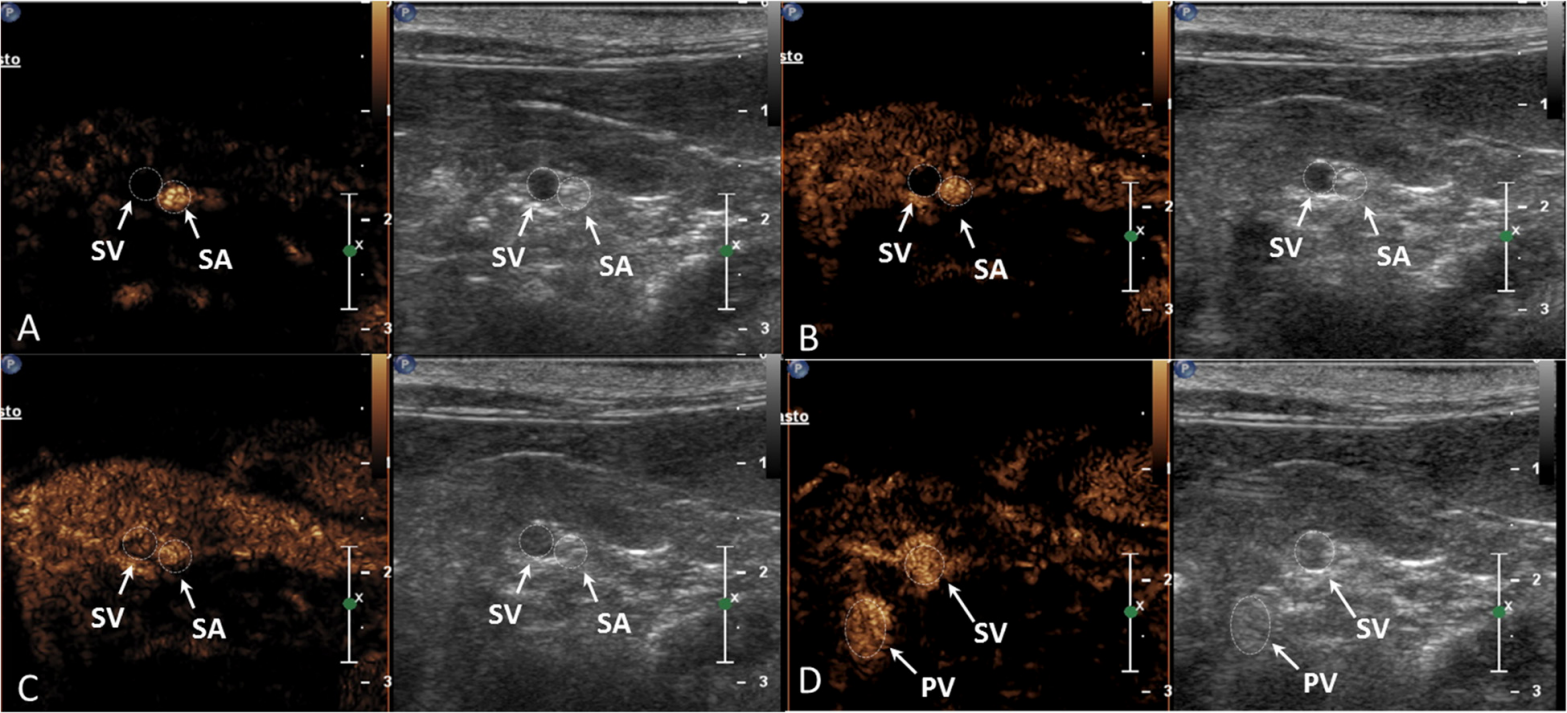
**A representative contrast-enhanced ultrasound sequence (A, B, C and D) of a normal left pancreatic lobe in a longitudinal plane after the injection of Sonovue.** Each image illustrates contrast enhancement on the left and the grey scale image on the right. **(A)** The contrast medium is visible in the splenic artery (SA). **(B)** Microbubbles are still visible in the splenic artery while the pancreatic parenchyma is starting to also receive some contrast material. The splenic vein appears anechoic on the contrast-enhanced image. **(C)** Homogeneous enhancement of the entire pancreatic lobe at peak enhancement. Some microbubbles are starting to fill the splenic vein (SV). **(D)** During the wash-out phase, the parenchyma progressively becomes hypoechoic, while the pancreatic vein (SV) reaches its peak in contrast uptake. The contrast medium is also visible in the portal vein (PV).

The pancreatic body and left lobe were identified in all cats. The margins of the pancreas were clearly recognised in six cats, while in the remaining four cats they appeared ill-defined. No adverse effects were noticed in any of the cats during or after the injection of the contrast medium. Contrast enhancement was judged subjectively to be satisfactory in nine of the ten cats. In one cat, the contrast uptake of the pancreas was considered insufficient and this cat was excluded from quantitative analyses. Following contrast medium administration, microbubbles were first seen in the splenic artery dorsally or caudo-dorsally to the left pancreatic lobe. This was followed by inflow of the contrast medium into the capillary bed of the pancreatic parenchyma, providing a well-marginated and uniformly contrast-enhanced pancreas at PI. An anechoic non-contrast-enhanced tubular or round structure (i.e., the splenic vein) was visualised immediately adjacent to the splenic artery in seven cats. During the wash-out period, the echogenicity of the pancreatic parenchyma reduced progressively, while the pancreatic artery showed residual intravascular contrast, which was subjectively judged to be less intense compared to the previous phases. The microbubble replenishment of the splenic and portal vein started at the PI of the pancreatic parenchyma and increased progressively during the wash-out phase, reaching a saturation of the signal. During PI, the pancreatic margins were always sharp and well-defined. This perfusion pattern of the left pancreatic lobe was consistently observed in all ten cats (Figure 3). In particular, replenishments of the splenic artery, splenic vein and portal vein were observed in ten, seven and ten cats, respectively. Quantitative computerised analysis of enhancement of the pancreatic body and left lobe was performed in nine cats and is summarised in Table 1.

**Table 1:** **Results of quantitative contrast-enhanced ultrasonography of the pancreatic body and left lobe in nine healthy cats**

**Parameter**	**Mean**	**SD**
AT (s)	4.69	1.26
TTPinj (s)	7.52	1.88
PI (a.u.)	6.58	2.66
TTPinr (s)	2.84	0.88
Wi (a.u./s)	2.18	1.79

AT, arrival time; TTPinj, time to peak from injection; PI, peak intensity; TTPinr, time to peak from initial rise; Wi, wash-in rate.

Measurements of the left pancreatic lobe, obtained from the grey scale image before contrast medium injection and from the contrast image at PI, are summarised in Table 2. There was a statistically significant difference between the thickness of the left pancreatic lobe before and after contrast medium injection (*P* < 0.0001).

**Table 2:** **Left pancreatic lobe thickness measured during grey scale analysis and contrast-enhanced ultrasound in ten healthy cats**

**Cat**	**Left pancreatic lobe thickness**	
	**Grey scale (mm)**	**Contrast enhancement (mm)**
1	5.3	5.8
2	5.2	6.5
3	7.7	8.7
4	5.8	6.5
5	5.4	5.4
6	6.6	7
7	4.2	4.7
8	4.6	5.5
9	5.7	6.6
10	4	5.5
Mean	5.5	6.2
SD	1.1	1.1

## Discussion

**Figure 4:**
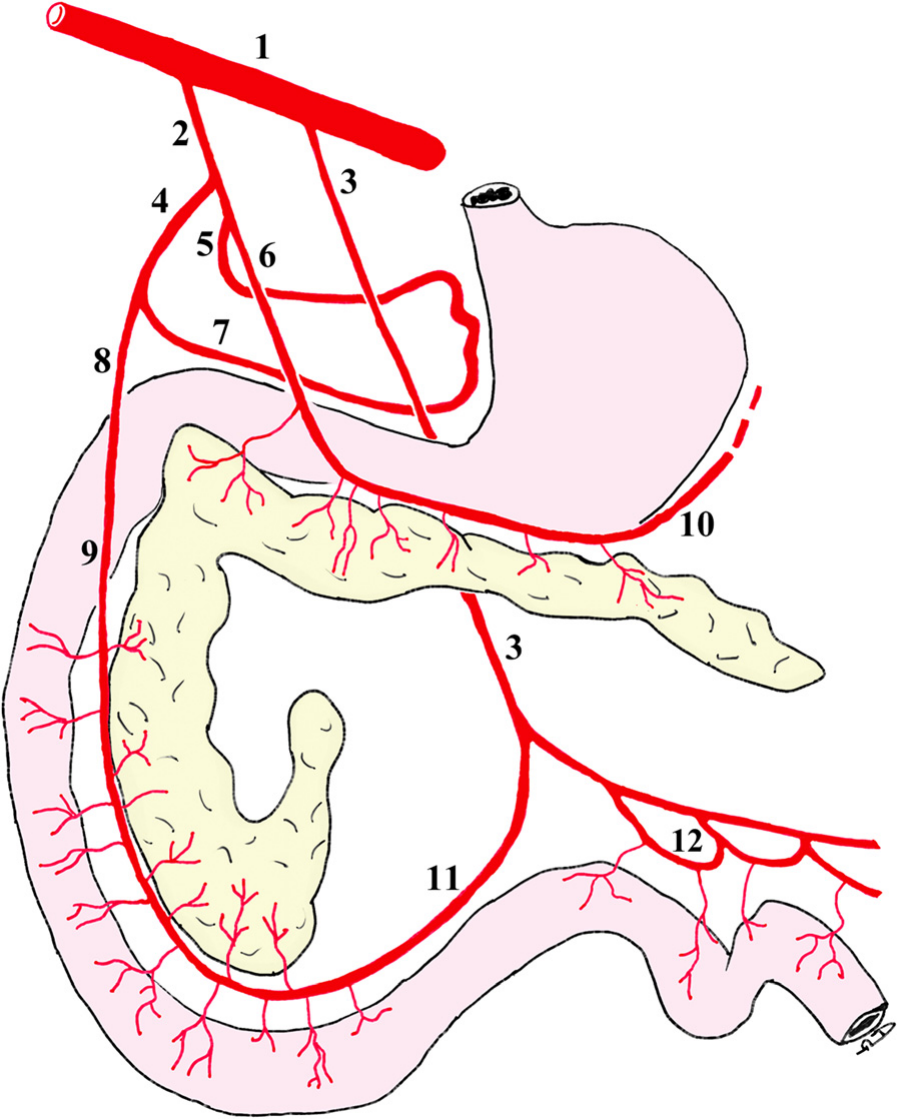
**Diagram of the distribution of the branches of the coeliac a. and cranial mesenteric a. to the pancreas.** 1, aorta; 2, coeliac a.; 3, cranial mesenteric a.; 4, hepatic a.; 5, left gastric a.; 6, splenic a.; 7, right gastric a.; 8, gastroduodenal a.; 9, cranial pancreaticoduodenal a.; 10, left gastroepiploic a.; 11, caudal pancreaticoduodenal a.; 12, jejunal aa.

Arterial blood flow is supplied to the pancreas by the splenic artery, the hepatic artery and the caudal pancreaticoduodenal artery. In particular, the left lobe, which is located parallel to the greater curvature of the stomach, is supplied by the splenic artery. This artery is the left branch of the coeliac artery; it runs in the greater omentum along the dorsocaudal surface of the left lobe of the pancreas [25] and gives off between two to six pancreatic branches for the left pancreatic lobe [26]. In addition to this main vasculature, the left pancreatic lobe is also supplied by a few small branches of the gastroduodenal artery (which is one of the two terminal endings of the hepatic artery) and, rarely, by a long branch of the caudal pancreaticoduodenal artery [26]. The body of the pancreas is supplied by one to three very small branches from the origin of the right gastric artery (which is the right branch of the coeliac artery) and from the gastroduodenal artery [27] or one of its terminal branches, the right gastroepiploic artery [26]. Tributaries of the major arteries of the pancreas penetrate the pancreatic parenchyma and arborise into one of three types of terminal afferent arterioles: capillary arterioles that surround and supply the exocrine pancreatic acini (i.e., acinar arterioles), capillary arterioles that supply the ductal system, and afferent arterioles that supply the capillary glomerulus of the islets of Langerhans (i.e., insular arterioles) [25-27]. A detailed description of the pancreatic vascular distribution is shown in Figure 4.

All cats showed a contrast-enhanced ultrasonographic pattern that followed the normal anatomic distribution of the vasculature described above. After the administration of the microbubble contrast agent, the splenic artery filled up rapidly. The intense and homogenous enhancement of the pancreatic parenchyma depends on small pancreatic branches of the splenic artery that arborise into a dense capillary network inside the pancreatic lobe. The wash-out phase was characterised by a gradual decrease of contrast medium within the pancreatic parenchyma, and an increase of contrast medium within the splenic and portal veins. This perfusion pattern is quite similar to that reported in previous CEUS studies performed in dogs [21,22], although those studies only evaluated the right pancreatic lobe and the adjacent duodenum. As described above, the blood supply to the right pancreatic lobe is different from that to the left lobe and depends on the cranial and caudal pancreaticoduodenal arteries that anastomose within the gland [26,27]. In a previous study conducted in healthy cats, a specific pattern of pancreatic perfusion was not described, as the author reported that the quantitative contrast enhanced the ultrasonographic analysis of perfusion in abdominal organs, including the pancreas [20]. In our study, good quality time-intensity curves were obtained for nine cats. In one cat, incorrect positioning of the intravenous catheter caused inadequate concentration of the contrast medium. Comparing our results of quantitative perfusion variables to those previously reported for healthy anaesthetised cats [20], the AT was similar to this previous study, while TTPinj and TTPinr were slightly higher in our cats. These results were expected, since several factors can influence quantitative CEUS parameters. For example, the study by Leinonen et al. employed general anaesthesia, and it has been previously demonstrated that different anaesthetic agents can induce cardiovascular changes which may influence contrast medium dynamics and markedly affect microbubble distribution and delivery [28]. Sedation and anaesthesia were avoided purposely in our cats, in order to obtain baseline values without iatrogenic changes in blood pressure and heart rate. Secondly, our analyses were performed at the level of the left pancreatic lobe, whereas Leinonen et al. did not specify the location for ROI measurements in the pancreatic parenchyma [20]. As previously mentioned, the pancreatic blood supply is quite different between the left and the right lobes. Several other factors, such as technical variables (e.g., gain setting, mechanical index and/or scanning depth), contrast agent type and dose, injection technique (bolus vs. constant rate infusion) and patient-related factors (e.g., heart rate, blood pressure and/or respiratory rate) can influence quantitative variables [28,29].

In the present study, we tried to reduce the technical variables as much as possible, keeping the same contrast ultrasound settings for all cats. A single 0.5 mL bolus dose of microbubble contrast medium was used for each cat. This dose was chosen on the basis of a previous study conducted by our group for the ultrasonographic evaluation of the small bowel using microbubble contrast medium in healthy cats [30].

The right lateral or dorsal recumbency was successfully used to evaluate the left pancreatic lobe, ventrally and medially to the spleen and the portal vein in the same image.

The edges of the left pancreatic lobe appeared ill-defined in four cats during B-mode ultrasonography, while they were clearly delineated from surrounding organs in contrast images for all cats. Furthermore, a statistical difference of the thickness of the left pancreatic lobe when measured from the grey scale image or the contrast-enhanced image was found. This finding can be explained on the basis of the abovementioned increased accuracy in defining pancreatic margins once the contrast medium has been injected. Pancreatic dimensions may not be correctly measured in grey scale images since peripancreatic fat has a similar echogenicity to the pancreatic parenchyma. This is unlikely to occur in contrast-enhanced images since the peritoneal fat doesn’t show contrast uptake, while the pancreatic parenchyma is highlighted by the microbubbles upon a dark background. This should be considered when accuracy is needed to measure pancreatic dimensions and outline its boundaries.

Like the other ultrasonographic techniques (i.e., B-mode sonography and Doppler sonography), CEUS is susceptible to some artefacts creating misinterpretation [31-33]. In particular, the slice thickness and the pseudoenhancement artefacts can produce an overestimation of the true perfused areas [31-33]. However, these artefacts occur in specific conditions such as deep region of investigation and low emission frequency probes [32,33]. The anatomical position of the feline pancreas, associated with the high frequency probe used in this study, makes an overestimation of the pancreatic measurements unlikely.

This study has some limitations that need to be emphasised. First, the population of cats prospectively recruited was considered healthy only on the basis of clinical and laboratory findings. No surgical biopsies were taken and, therefore, histological confirmation of the normality of the pancreas was not available. Second, the CEUS was performed using a single bolus of contrast medium and directed only at the left pancreatic lobe. A recent report demonstrated that continuous infusion of contrast medium provided better detail of the vascular distribution as well as a prolonged enhancement of the pancreas, which was determined to be useful in detecting differences in pancreatic perfusion during diffuse disease [23].

## Conclusions

In conclusion, we demonstrated that CEUS can be used for evaluation of the pancreatic parenchyma in healthy cats. In particular, the injection of a microbubble contrast medium allows a precise definition of pancreatic edges. A distinctive contrast perfusion pattern is described. The baseline data described here may be useful as a reference for future assessment of cats with pancreatic disease.

## Endnotes

^a^iU22 ultrasound system, Philips Healthcare, Monza, Italy.

^b^Sonovue, Bracco® diagnostic, Milano, Italy.

^c^Showcase software, Trillium Technology, Ann Arbor, MI.

^d^QLAB quantification software, Philips Healthcare, Monza, Italy.

^e^Prism 5®, GraphPad Software Inc., San Diego, CA.

## Abbreviations

AT: Arrival time
CEUS: Contrast-enhanced ultrasound
fPLI: Feline pancreatic lipase immunoreactivity
fTLI: Feline trypsin-like immunoreactivity
PI: Peak intensity
ROI: Region of interest
SD: Standard deviation
TTP inj: Time to peak from injection
TTP inr: Time to peak from initial rise
Wi: Wash-in rate
